# Gestodene Accelerates Cutaneous Wound Healing via PAR1-Selective Positive Allosteric Modulation

**DOI:** 10.3390/ijms27125502

**Published:** 2026-06-18

**Authors:** Hyejin Jeon, Yunkyung Heo, Yechan Lee, So-Hyeon Park, Mincheol Kang, Wan Namkung

**Affiliations:** 1 College of Pharmacy and Yonsei Institute of Pharmaceutical Sciences, Yonsei University, 85 Songdogwahak-ro, Yeonsu-gu, Incheon 21983, Republic of Korea; isy0803@yonsei.ac.kr (H.J.); ykheo107@naver.com (Y.H.); llyycc94@naver.com (Y.L.); sohyeon0605@yonsei.ac.kr (S.-H.P.); alscjf1592@naver.com (M.K.); 2 Woo Choo Lee Institute for Precision Drug Development, Yonsei University College of Medicine, Seoul 03722, Republic of Korea

**Keywords:** PAR1, positive allosteric modulator, proliferation, migration, wound repair

## Abstract

Protease-activated receptor 1 (PAR1), a G protein-coupled receptor, plays a central role in coordinating multiple phases of cutaneous wound healing, including hemostasis, cell proliferation, migration, and extracellular matrix remodeling. Despite its therapeutic potential, PAR1-selective positive allosteric modulators (PAMs) remain limited. Here, we characterized the wound healing efficacy of gestodene, a third-generation progestin previously identified as a selective PAM of PAR1. Gestodene exhibited no intrinsic agonist activity but selectively potentiated PAR1-activating peptide (PAR1-AP)-induced calcium signaling without affecting PAR2 or PAR4 responses. Consistently, gestodene induced a concentration-dependent leftward shift in the PAR1-AP dose–response curve. Notably, gestodene enhanced PAR1-dependent cell proliferation, migration, and ERK1/2 activation, effects abolished by PAR1 knockout or pharmacological inhibition with vorapaxar in human keratinocytes (HaCaT) and dermal fibroblasts (HDF). Gestodene also potentiated the expression of wound healing-associated genes, including matrix metalloproteinases (MMP-1, -2, -3, -10), fibronectin, and type I collagen (COL1A1). In a murine wound model, topical administration of gestodene accelerated wound closure, achieving complete re-epithelialization by Day 8 and significantly enhancing collagen deposition, effects reversed by vorapaxar. Collectively, these findings demonstrate that gestodene accelerates cutaneous wound healing through PAR1-selective positive allosteric modulation and supports its potential as a drug repositioning candidate for wound repair.

## 1. Introduction

Wound healing is a complex and dynamic biological process essential for the restoration of tissue integrity following injury. This multi-phase process encompasses hemostasis, inflammation, proliferation, and extracellular matrix remodeling, culminating in re-epithelialization. In adult tissues, wound repair is initiated by rapid hemostasis, followed by a tightly regulated inflammatory response, mesenchymal cell activation and migration, angiogenesis, and epithelial regeneration [1,2].

Chronic and non-healing wounds represent a major global health burden, affecting millions of patients and imposing substantial economic costs on healthcare systems [3,4,5]. Despite advances in antiseptics and antimicrobial therapies, current treatments often fail to precisely regulate the complex signaling networks that coordinate each phase of wound repair [6,7,8]. These limitations highlight the need for novel pharmacological strategies capable of orchestrating cellular and molecular events during tissue regeneration.

Protease-activated receptor 1 (PAR1), a G protein-coupled receptor (GPCR), has emerged as a central regulator of wound healing [9]. PAR1 is activated through proteolytic cleavage of its N-terminus by multiple proteases, including thrombin, activated protein C (APC), meizothrombin, plasmin, MMP-1, and cathepsin G [10,11,12,13]. Upon activation, PAR1 initiates downstream signaling pathways such as mitogen-activated protein kinase (MAPK) and phosphoinositide 3-kinase (PI3K), which promote cell survival, proliferation, and migration [14,15]. In keratinocytes and fibroblasts, PAR1 signaling drives the expression of wound healing-associated genes, including matrix metalloproteinases (MMPs), fibronectin, and collagen, key mediators of extracellular matrix remodeling [16,17]. Collectively, these findings position PAR1 as a key integrator of hemostasis, cellular responses, and matrix remodeling during wound repair.

Despite its therapeutic promise, pharmacological targeting of PAR1 remains challenging. While endogenous activation by proteases is essential for physiological wound healing, exogenous PAR1-activating peptides (PAR1-APs) induce maximal receptor stimulation, which can lead to receptor desensitization and dysregulated coagulation signaling [18,19,20]. In contrast, positive allosteric modulators (PAMs) enhance receptor responsiveness to endogenous ligands without directly activating the receptor, providing a more physiologically relevant approach [21,22]. Although GB83 has been reported as a PAR1 PAM with in vivo wound healing activity, its lack of selectivity due to concomitant modulation of PAR2 limits its therapeutic utility [16,23,24]. Therefore, the development of PAR1-selective PAMs remains an important unmet need.

Gestodene has recently been identified as a PAR1-selective PAM that enhances receptor signaling without intrinsic agonist activity [25]. In this study, we investigated the therapeutic potential of gestodene in wound healing. We evaluated its effects on PAR1-mediated cellular responses in human keratinocytes (HaCaT) and dermal fibroblasts (HDF), confirmed PAR1 dependency using genetic and pharmacological approaches, and assessed its efficacy in a murine wound healing model.

## 2. Results

### 2.1. Characterization of Positive Allosteric Modulator of PAR1, Gestodene

**Figure 1:**
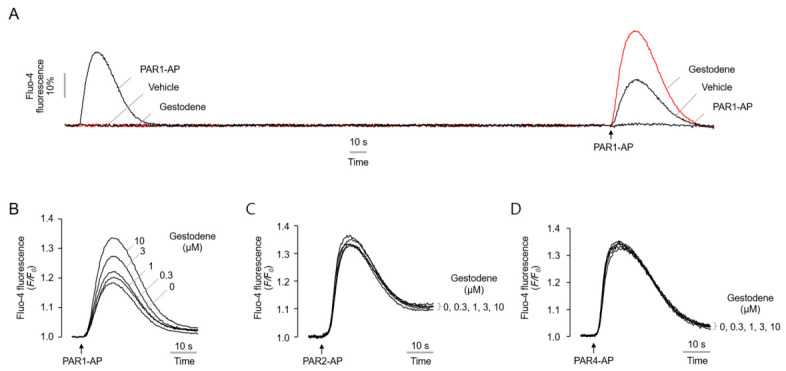
Effect of gestodene on PAR1-AP-, PAR2-AP-, and PAR4-AP-induced intracellular Ca^2+^ signaling in HT29 cells. (**A**) Gestodene (10 µM, red), vehicle (DMSO, black), or PAR1-AP (20 µM, gray) was applied 5 min prior to PAR1-AP (10 µM) treatment. Gestodene enhanced PAR1-AP-induced Ca^2+^ signaling without directly activating PAR1, whereas pre-exposure to PAR1-AP itself induced rapid homologous desensitization, abolishing the response to subsequent PAR1-AP challenge. (**B**–**D**) Concentration-dependent potentiation of PAR-AP-induced Ca^2+^ responses by gestodene. Cells were pretreated with gestodene at the indicated concentrations for 10 min prior to stimulation with (**B**) PAR1-AP (10 µM), (**C**) PAR2-AP (10 µM), or (**D**) PAR4-AP (50 µM).

In our previous study, gestodene was identified as a novel PAM of PAR1 [25]. Consistent with this, gestodene alone (10 μM) did not induce intracellular calcium mobilization in HT29 cells, indicating a lack of intrinsic agonist activity. However, gestodene significantly potentiated PAR1-AP-induced calcium responses. This effect was selective for PAR1, as gestodene enhanced PAR1-mediated signaling without affecting PAR2- or PAR4-mediated responses (Figure 1A–D).

**Figure 2:**
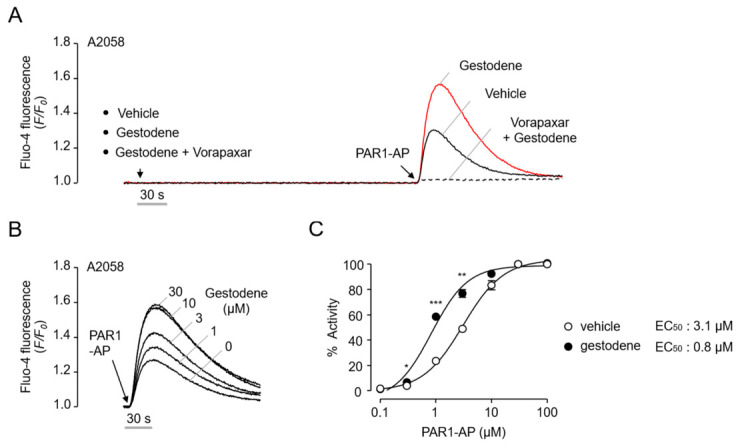
Enhancement of PAR1-AP-induced intracellular calcium signaling by gestodene in A2058 cells. (**A**) PAR1 was partially activated by 1 μM PAR1-AP in the presence or absence of gestodene. Gestodene (10 μM) or vehicle (DMSO) was applied 10 min prior to PAR1-AP treatment. PAR1 was blocked by 100 nM vorapaxar, a selective PAR1 antagonist. (**B**) The indicated concentrations of gestodene were applied 10 min prior to partial activation of PAR1 by PAR1-AP in A2058 cells. (**C**) Dose–response curve for PAR1-AP-induced intracellular calcium mobilization in A2058 cells in the absence and presence of gestodene (mean ± S.E., *n* = 4). * *p* < 0.05, ** *p* < 0.01, *** *p* < 0.001 versus vehicle control.

To further characterize the PAR1 modulation, gestodene was evaluated in A2058 cells, a human melanoma cell line. In A2058 cells, gestodene significantly potentiated calcium responses elicited by suboptimal concentrations of PAR1-AP (Figure 2A). This potentiation was completely abolished by vorapaxar, confirming a PAR1-dependent mechanism. Consistent with allosteric modulation, gestodene induced a pronounced leftward shift in the PAR1-AP dose–response curve, decreasing the EC_50_ from 3.1 ± 0.03 μM to 0.8 ± 0.05 μM (~4-fold increase in sensitivity) (Figure 2B,C).

**Figure 3:**
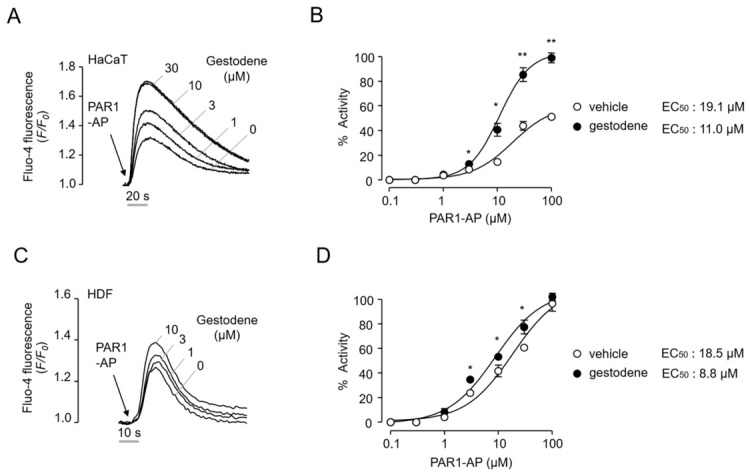
Enhancement of PAR1-AP-induced intracellular calcium signaling by gestodene in HaCaT and HDF cells. (**A**) The indicated concentrations of gestodene were applied 10 min prior to partial activation of PAR1 in HaCaT cells. (**B**) Dose–response curve for PAR1-AP-induced intracellular calcium mobilization in HaCaT cells in the absence and presence of gestodene (mean ± S.D., *n* = 4). (**C**) The indicated concentrations of gestodene were applied 10 min prior to partial activation of PAR1 in HDF cells. (**D**) Dose–response curve for PAR1-AP-induced intracellular calcium mobilization in HDF cells in the absence and presence of gestodene (mean ± S.D., *n* = 4). * *p* < 0.05, ** *p* < 0.01.

In both human keratinocytes (HaCaT) and dermal fibroblasts (HDF), gestodene concentration-dependently potentiated PAR1-AP-induced calcium mobilization. Consistent with its allosteric mechanism, gestodene induced marked leftward shifts in the PAR1-AP dose–response curves, indicating increased PAR1 receptor sensitivity (Figure 3A–D). To further assess the activity of gestodene in the context of physiologically relevant PAR1 activation, the effect of gestodene on thrombin-induced calcium signaling was evaluated in HaCaT cells. Gestodene concentration-dependently potentiated thrombin (0.4 unit/mL)-induced intracellular calcium mobilization, consistent with its PAM activity observed with PAR1-AP (Figure S1).

### 2.2. Gestodene Accelerates Cell Proliferation and Migration of A2058, HaCaT and HDF Cells in a PAR1-Dependent Manner

**Figure 4:**
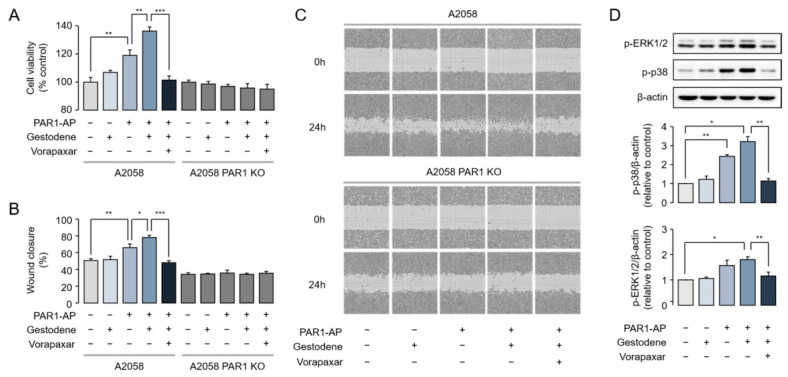
Enhancement of PAR1-stimulated cell proliferation and migration by gestodene in A2058 cells. (**A**) Effect of gestodene on PAR1-mediated increases in cell proliferation. A2058 and PAR1 knockout (KO) cells were treated with gestodene (3 μM), PAR1-AP (10 μM), and vorapaxar (30 nM) for 3 days, and cell viability was measured using an MTS assay. Cells were maintained in serum-free medium (mean ± S.E., *n* = 5). (**B**,**C**) Effect of gestodene on PAR1-mediated increases in cell migration. An in vitro wound healing assay was performed in A2058 and PAR1 KO cells. Cells were treated with gestodene (3 μM), PAR1-AP (10 μM), and vorapaxar (30 nM). Representative images were taken at 0 and 24 h post-wounding. Wound closure was quantified every 4 h post-wounding (mean ± S.E., *n* = 3). (**D**) Effect of gestodene on phospho-ERK1/2, phospho-p38, and β-actin expression. A2058 cells were incubated with gestodene (10 μM) with or without vorapaxar (100 nM) for 30 min in serum-free culture medium and then stimulated with PAR1-AP (10 μM) for 5 min. Western blot analysis was performed, and densitometric quantification was normalized to β-actin (mean ± S.E., *n* = 3). * *p* < 0.05, ** *p* < 0.01, *** *p* < 0.001.

To establish the PAR1 dependency of the potentiation effects of gestodene, cell proliferation and migration were assessed in wild-type and PAR1 knockout A2058 cells. A2058 cells express high levels of PAR1, enabling direct comparison between wild-type and PAR1-deficient cells [26]. All experiments were conducted in serum-free medium to avoid interference from thrombin present in fetal bovine serum. In wild-type A2058 cells, gestodene significantly potentiated PAR1-AP-induced cell proliferation and migration compared to PAR1-AP alone (Figure 4A–C). Notably, this potentiation effect was markedly attenuated in PAR1 knockout cells, and pharmacological blockade with vorapaxar abolished these effects, confirming that the observed responses are mediated through PAR1. In addition, gestodene enhanced PAR1-AP-induced phosphorylation of ERK1/2 and p38 MAPK in A2058 cells (Figure 4D).

**Figure 5:**
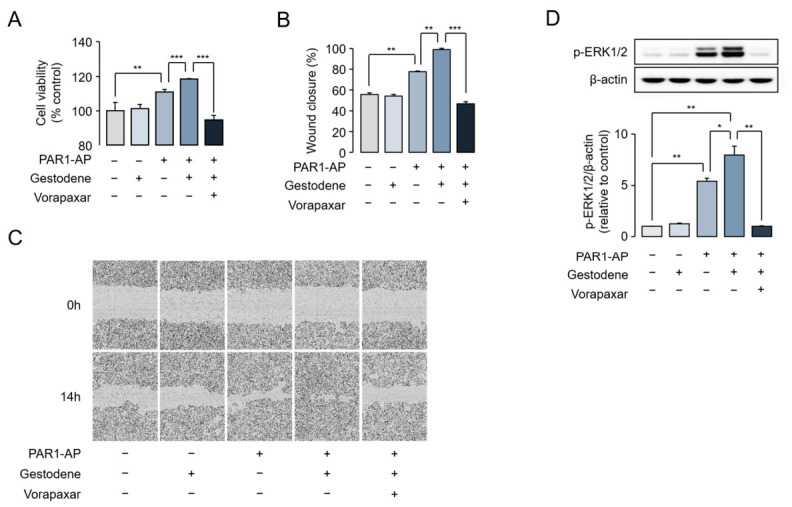
Enhancement of PAR1-stimulated cell proliferation and migration by gestodene in HaCaT cells. (**A**) Effect of gestodene on PAR1-mediated increases in cell viability. HaCaT cells were treated with gestodene (3 μM), PAR1-AP (10 μM), and vorapaxar (30 nM) for 3 days, and cell viability was measured using an MTS assay. Cells were maintained in serum-free medium (mean ± S.E., *n* = 5). (**B**,**C**) Effect of gestodene on PAR1-mediated increases in cell migration. An in vitro wound healing assay was performed in HaCaT cells. Cells were treated with gestodene (3 μM), PAR1-AP (10 μM), and vorapaxar (30 nM). Representative images were taken at 0 and 14 h post-wounding. Wound closure was quantified every 2 h post-wounding (mean ± S.E., *n* = 3). (**D**) Effect of gestodene on phospho-ERK1/2 and β-actin expression. HaCaT cells were incubated with gestodene (10 μM) with or without vorapaxar (100 nM) for 30 min in serum-free culture medium and then stimulated with PAR1-AP (10 μM) for 5 min. Western blot analysis was performed, and densitometric quantification was normalized to β-actin (mean ± S.E., *n* = 3). * *p* < 0.05, ** *p* < 0.01, *** *p* < 0.001.

Having established PAR1 dependency in A2058 cells, the effects of gestodene were further evaluated in wound healing-relevant skin cells. In HaCaT cells, gestodene potentiated PAR1-AP-induced cell proliferation and migration, and these effects were abolished by vorapaxar (Figure 5A–C). Gestodene also enhanced PAR1-AP-induced phosphorylation of ERK1/2 in HaCaT cells (Figure 5D).

**Figure 6:**
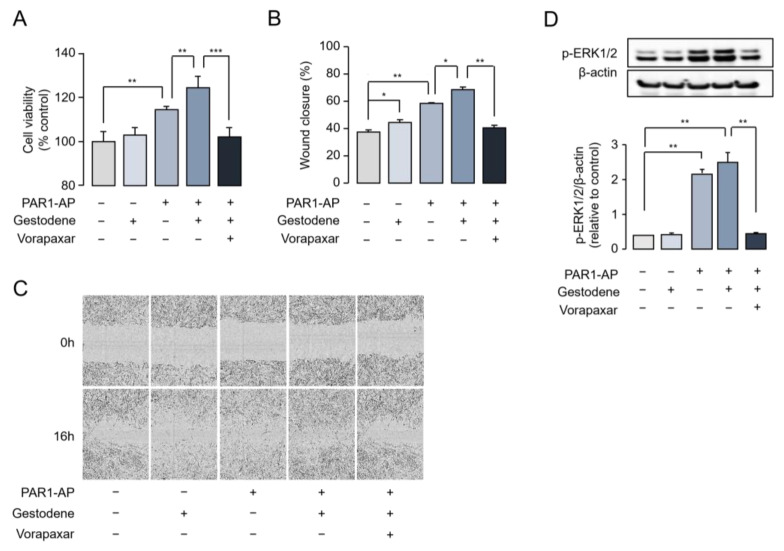
Enhancement of PAR1-stimulated cell proliferation and migration by gestodene in HDF cells. (**A**) HDF cells were treated with gestodene (3 μM), PAR1-AP (10 μM), and vorapaxar (30 nM) for 3 days, and cell proliferation was measured using an MTS assay. Cells were maintained in serum-free medium (mean ± S.E., *n* = 5). (**B**,**C**) Effect of gestodene on PAR1-mediated increases in cell migration. An in vitro wound healing assay was performed in HDF cells. Cells were treated with gestodene (3 μM), PAR1-AP (10 μM), and vorapaxar (30 nM). Representative images were taken at 0 and 16 h post-wounding. Wound closure was quantified every 4 h post-wounding (mean ± S.E., *n* = 3). (**D**) Effect of gestodene on phospho-ERK1/2 and β-actin expression. HDF cells were incubated with gestodene (10 μM) with or without vorapaxar (100 nM) for 30 min in serum-free culture medium and then stimulated with PAR1-AP (10 μM) for 5 min. Western blot analysis was performed, and densitometric quantification was normalized to β-actin (mean ± S.E., *n* = 3). * *p* < 0.05, ** *p* < 0.01, *** *p* < 0.001.

Similarly, in HDF cells, gestodene potentiated PAR1-AP-induced cell proliferation and migration, and vorapaxar blocked these potentiation effects (Figure 6A–C). Gestodene also enhanced PAR1-AP-induced ERK1/2 phosphorylation in HDF cells (Figure 6D), supporting its PAR1-dependent activity in multiple wound-relevant cell types.

### 2.3. Gestodene Promotes Gene Expression of MMP, Fibronectin and COL1A1 in HaCaT and NIH-3T3 Cells

**Figure 7:**
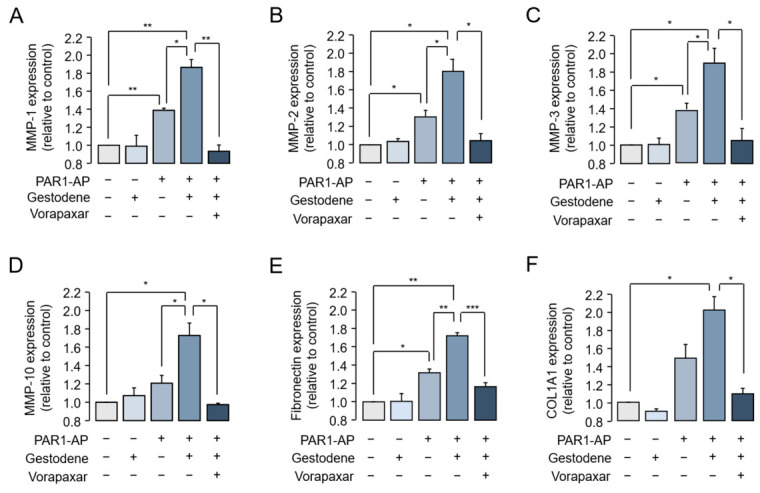
Enhancement of PAR1-stimulated expression of wound healing-related genes. (**A**–**E**) HaCaT cells were treated with PAR1-AP (10 μM), gestodene (3 μM), and vorapaxar (30 nM) as indicated for 24 h, and mRNA levels of MMPs and fibronectin were measured using quantitative real-time PCR. (**F**) NIH-3T3 cells were treated with PAR1-AP (10 μM), gestodene (3 μM), and vorapaxar (30 nM) as indicated for 24 h, and mRNA levels of COL1A1 were measured. All mRNA levels were normalized to β-actin (mean ± S.E., *n* = 3). * *p* < 0.05, ** *p* < 0.01, *** *p* < 0.001.

Matrix metalloproteinases (MMPs) play a critical role in extracellular matrix remodeling during wound healing [27,28]. To investigate whether gestodene modulates MMP expression, mRNA levels of MMP-1, MMP-2, MMP-3, and MMP-10 were examined in HaCaT cells. PAR1-AP treatment increased the expression of these MMPs, and gestodene further potentiated these PAR1-AP-induced increases (Figure 7A–D). These potentiation effects were abolished by vorapaxar, indicating that gestodene enhances MMP expression through a PAR1-dependent mechanism.

Fibronectin and type I collagen (encoded by COL1A1) are major extracellular matrix components essential for tissue repair and wound closure [29,30]. In HaCaT cells, PAR1-AP treatment increased fibronectin expression, and gestodene further potentiated this PAR1-AP-induced increase, an effect that was blocked by vorapaxar (Figure 7E). To assess collagen-associated gene expressions, COL1A1 mRNA levels were examined in NIH-3T3 fibroblasts, a commonly used model for collagen synthesis. PAR1-AP treatment increased COL1A1 expression in NIH-3T3 cells, and gestodene further potentiated this PAR1-AP-induced increase. This potentiation was also abolished by vorapaxar (Figure 7F), suggesting that gestodene enhances extracellular matrix-related gene expression via PAR1-dependent signaling.

### 2.4. Gestodene Enhances Skin Wound Healing Through PAR1-Mediated Activity in ICR Mice

To investigate whether gestodene promotes wound healing in vivo, full-thickness dorsal skin wounds were induced in ICR mice using sterile biopsy punches following hair removal, and the wound area was monitored over 8 days. Gestodene was topically administered at a dose of 2 mg/vaseline, while vorapaxar was applied at a dose of 0.6 mg/vaseline.

**Figure 8:**
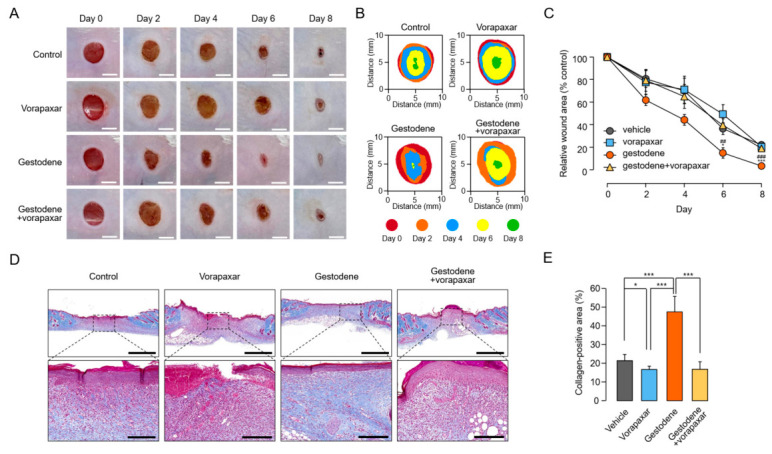
Effect of gestodene on skin wound closure in ICR mice. (**A**) Representative images of excisional wounds on dorsal skin. Seven-week-old ICR mice were treated daily with vehicle, vorapaxar, gestodene, or gestodene combined with vorapaxar for 8 days at the wound site. Scale bar = 0.5 cm. (**B**) Schematic representation of wound contraction over 8 days following treatment with vehicle, vorapaxar, gestodene, or gestodene combined with vorapaxar. (**C**) Relative wound area was quantified using ImageJ software (mean ± S.E.M., *n* = 6 per group). * *p* < 0.05 and *** *p* < 0.001 indicate comparisons versus vehicle, and ^##^ *p* < 0.05 and ^###^ *p* < 0.001 indicate comparisons between gestodene and gestodene + vorapaxar. (**D**) Representative images of Masson’s trichrome-stained tissue sections on Day 8. Scale bars = 1 mm (overview) and 0.2 mm (high-magnification inset). (**E**) Collagen-positive area was quantified using ImageJ software (mean ± S.E.M., *n* = 6 per group). Statistical analysis was performed using one-way ANOVA followed by Tukey’s post hoc test, as appropriate. Non-significant differences are not indicated. Exact *p* values for all pairwise comparisons at each time point (Days 2, 4, 6, and 8) are provided in Table S1.

Gestodene significantly accelerated wound closure compared to the vehicle control, and this effect was abolished by co-treatment with vorapaxar (Figure 8A–C), indicating a PAR1-dependent mechanism. By day 8, wounds treated with gestodene exhibited complete closure, whereas the control, vorapaxar, and combination treatment groups retained residual wound areas of 22.02%, 20.28%, and 18.94%, respectively, relative to the initial wound size, demonstrating a marked improvement in wound healing in the gestodene-treated group.

Histological analysis of wound tissues was performed using Masson’s trichrome staining at day 8 post-treatment to assess collagen deposition. The gestodene-treated group exhibited extensive collagen fiber accumulation, evidenced by prominent blue staining throughout the wound area (Figure 8D,E). In contrast, the control, vorapaxar, and combination treatment groups showed comparatively reduced collagen-positive areas, consistent with the observed differences in wound closure.

## 3. Discussion

Gestodene, originally developed as a third-generation progestin for contraceptive use, has recently been identified as a selective positive allosteric modulator (PAM) of PAR1 [25,31]. This finding highlights a drug repositioning opportunity, whereby an approved pharmaceutical agent can be applied to a new therapeutic indication. In this context, drug repositioning offers several advantages, including established safety profiles, known pharmacokinetics, and reduced development timelines compared to de novo drug discovery [32,33].

At the mechanistic level, in vitro characterization demonstrated that gestodene does not directly activate PAR1 but instead potentiates PAR1-mediated calcium signaling in a concentration-dependent manner. As reported previously, gestodene binds to a putative allosteric site distinct from the orthosteric binding pocket of PAR1, thereby enhancing receptor sensitivity to agonist stimulation without directly activating the receptor [25]. Importantly, gestodene had no effect on PAR2- or PAR4-mediated responses, confirming its identity as a PAR1-selective PAM (Figure 1). Consistent with this mechanism, gestodene induced a leftward shift in the PAR1-AP dose–response curve across multiple cell types, including wound-relevant HaCaT keratinocytes and HDF (Figure 2 and Figure 3), indicating enhanced receptor sensitivity to agonist stimulation. In addition, gestodene concentration-dependently potentiated thrombin-induced calcium signaling in HaCaT keratinocytes (Figure S1), further supporting its activity in the context of physiologically relevant protease-mediated PAR1 activation.

From a pharmacological perspective, this mode of action may provide advantages over direct PAR1 agonists, which typically induce maximal receptor activation followed by rapid desensitization [18,19,34]. In contrast, PAMs amplify endogenous signaling only in the presence of physiological agonists such as thrombin, thereby preserving the spatiotemporal regulation of receptor activation. As a result, this mechanism may reduce the risk of adverse effects associated with sustained or non-physiological receptor activation [16,35,36].

Another key feature of gestodene is its subtype selectivity. Unlike the previously reported compound GB83, which exhibits dual activity on PAR1 and PAR2 [16,23,24], gestodene selectively modulates PAR1 without affecting PAR2 or PAR4. Given that PAR family members mediate distinct biological functions in the skin, this selectivity is particularly important. PAR1 is primarily associated with platelet activation, endothelial barrier function, and fibroblast proliferation, all of which contribute to tissue repair. In contrast, PAR2 is more closely linked to inflammatory signaling, epithelial barrier disruption, and type 2 immune responses [37,38,39,40]. Therefore, non-selective modulation of multiple PAR subtypes may lead to conflicting biological outcomes, especially in chronic wounds characterized by dysregulated inflammation. In this regard, the selective potentiation of PAR1 by gestodene represents a more targeted pharmacological strategy. Furthermore, direct comparison confirmed that gestodene produced markedly greater potentiation of PAR1-AP-induced calcium signaling than GB83, with increases of approximately 124% and 64% above PAR1-AP alone, respectively (Figure S2A,B). Regarding maximal receptor activation, GB83 failed to significantly increase Emax under saturating conditions (Figure S2C), whereas gestodene increased the Emax by approximately 2-fold compared to PAR1-AP alone (Figure 3B).

Consistent with its pharmacological profile, gestodene enhanced PAR1-dependent cell proliferation, migration, and ERK1/2 activation across multiple cell types (Figure 4, Figure 5 and Figure 6). The PAR1 dependency of these effects was supported by both genetic and pharmacological evidence. In A2058 cells, PAR1 knockout markedly attenuated the proliferative and migratory responses to gestodene, providing genetic validation of receptor specificity (Figure 4). In parallel, pharmacological inhibition with vorapaxar abolished these effects across all tested cell types. Notably, these findings were reproduced in HaCaT and HDF cells, which are key effector cells in cutaneous wound healing, thereby reinforcing the physiological relevance of the observed mechanisms (Figure 5 and Figure 6).

At the transcriptional level, gestodene promoted PAR1-dependent upregulation of genes associated with extracellular matrix remodeling, including MMPs, fibronectin, and collagen (Figure 7). These transcriptional responses play a critical role in coordinating tissue remodeling and wound closure. Taken together, these findings indicate that gestodene modulates multiple stages of the wound-healing process, ranging from early signaling events such as calcium mobilization and MAPK activation to downstream transcriptional programs involved in extracellular matrix remodeling. However, because MMP expression and collagen/fibronectin induction were assessed at representative time points rather than through a comprehensive time-course analysis, the temporal relationship between matrix degradation and matrix deposition cannot be definitively established from the current data. In addition, although the findings support a role for gestodene in extracellular matrix remodeling, collagen expression was evaluated using NIH-3T3 cells, which may not fully recapitulate the responses of human dermal fibroblasts.

Consistent with these findings, the in vitro effects of gestodene were recapitulated in vivo. In a murine skin wound model, topical administration of gestodene accelerated wound closure and increased collagen deposition in a PAR1-dependent manner, as evidenced by the inhibitory effect of vorapaxar (Figure 8). Collectively, these results provide in vivo support for the proposed mechanism and demonstrate that the cellular and molecular effects of gestodene translate into functional tissue repair outcomes. However, because this study focused on an acute wound model, the long-term consequences of sustained PAR1 potentiation remain unclear. In particular, whether enhanced collagen accumulation may influence scar quality, excessive matrix deposition, or fibrotic remodeling in chronic wound settings warrants further investigation. In addition, the micromolar concentrations of gestodene used in this study exceed typical systemic exposure levels during oral contraceptive use [31]. Therefore, potential off-target effects, including interactions with progesterone receptors or other nuclear hormone receptors, cannot be fully excluded under local topical exposure conditions.

In conclusion, gestodene acts as a PAR1-selective positive allosteric modulator that enhances multiple components of the wound healing process in vitro and promotes tissue repair in vivo. Its subtype selectivity, combined with an agonist-dependent mechanism of action, distinguishes it from previously described PAR modulators and may offer a more controlled and physiologically relevant therapeutic approach. Taken together, these findings support the potential of gestodene as a drug repositioning candidate for wound healing and provide a rationale for further development of PAR1-selective PAMs as therapeutic agents for tissue repair.

## 4. Materials and Methods

### 4.1. Cell Culture and Cell Lines

A2058 (RRID: CVCL_1059), HaCaT (RRID: CVCL_0038), NIH-3T3 (RRID: CVCL_0594), and HT29 (RRID: CVCL_0320) cells were obtained from the Korean Cell Line Bank (Seoul, Republic of Korea). HDF cells were kindly provided by Dr. Jong-Hyuk Sung (Yonsei University). Cells were maintained at 37 °C and 5% CO_2_. A2058, HaCaT, NIH-3T3, and HDF cells were cultured in Dulbecco’s modified Eagle medium (DMEM), while HT29 cells were cultured in Roswell Park Memorial Institute (RPMI) 1640 medium. All media were supplemented with 10% fetal bovine serum (FBS), 100 units/mL penicillin, and 100 μg/mL streptomycin.

### 4.2. Materials and Reagents

Thrombin, ATP, and gestodene were purchased from Sigma-Aldrich (St. Louis, MO, USA), vorapaxar from Tocris Bioscience (Atlantic Road, Bristol, UK). PAR1-AP (TFLLRN-NH2) was synthesized from Cosmogenetech Co., Ltd. (Seoul, Republic of Korea).

### 4.3. Intracellular Calcium Measurement

A2058, HaCaT, HDF, and HT29 cells were seeded overnight in 96-well clear-bottom, black-walled plates (Corning Inc., Corning, NY, USA). Cells were then loaded with Fluo-4 NW fluorescent dye (Invitrogen, Carlsbad, CA, USA) and allowed to incubate for 1 h following the manufacturer’s protocol. Compounds were applied 10 min prior to agonist addition, and PAR1-AP was introduced 5 s after the onset of fluorescence recording. Fluorescence signals were detected using a FLUOstar Omega microplate reader (BMG LABTECH, Offenburg, Germany).

### 4.4. Cell Proliferation Assay

Cell proliferation was assessed using the CellTiter 96^®^ AQueous One Solution Cell Proliferation Assay kit (MTS; Promega, Madison, WI, USA). A2058, HaCaT, and HDF cells were plated in 96-well plates with growth medium containing 10% FBS and cultured for 24 h. Cells were subsequently washed twice with PBS and the medium was replaced with serum-free medium. Gestodene, PAR1-AP, and vorapaxar were administered at 24 h intervals, with an equivalent volume of DMSO serving as the vehicle control. Following 72 h of treatment, the medium was removed and the assay was carried out according to the manufacturer’s instructions. Absorbance was recorded at 490 nm with a reference wavelength of 690 nm using an Infinite M200 microplate reader (Tecan, Männedorf, Switzerland).

### 4.5. In Vitro Scratch Wound Healing Assay

The effect of gestodene on cell migration was assessed by an in vitro scratch wound healing assay. A2058, HaCaT, and HDF cells were grown to approximately 100% confluency to establish a uniform monolayer in 96-well plates. Following scratch formation, cells were rinsed twice with PBS and subsequently treated with 200 µL of serum-free DMEM containing gestodene, PAR1-AP, vorapaxar, or DMSO as a vehicle control. Wound images were acquired at defined intervals using an IncuCyte ZOOM live-cell imaging system (Essen BioScience, Ann Arbor, MI, USA), and the degree of wound closure was quantified using the accompanying IncuCyte analysis software 2018A. The time points selected for representative imaging (14 h for HaCaT, 16 h for HDF, and 24 h for A2058 cells) were determined to optimally distinguish treatment group differences across independent experiments.

### 4.6. Western Blot Analysis

For immunoblot analysis, A2058 and HaCaT cells were rinsed twice with ice-cold PBS, then lysed in cold RIPA buffer containing a protease inhibitor cocktail for 15 min, followed by centrifugation at 13,000 rpm for 20 min. Protein concentrations were determined using a Bradford protein assay kit (Thermo Scientific, Waltham, MA, USA). Equal amounts of protein (30 µg) were resolved by 4–12% Tris-glycine precast gel electrophoresis (Koma Biotech, Seoul, Republic of Korea) and subsequently transferred onto PVDF membranes (Millipore, Billerica, MA, USA). Membranes were blocked for 1 h with 5% BSA in TBST, then incubated overnight at 4 °C with the following primary antibodies: anti-phospho-p42/44 (Cat. No. 9101; Cell Signaling Technology, Danvers, MA, USA), anti-phospho-p38 (Cat. No. sc-166182; Santa Cruz Biotechnologies, Santa Cruz, CA, USA), and anti-β-actin (Cat. No. sc-47778; Santa Cruz Biotechnologies). After three washes in TBST (5 min each), membranes were incubated with HRP-conjugated secondary antibodies for 1 h and visualized using the ECL Plus immunoblotting detection system (GE Healthcare, Piscataway, NJ, USA). Band intensities were quantified using ImageJ software (version 1.53e, NIH, Bethesda, MD, USA).

### 4.7. Quantitative Real-Time PCR

Total mRNA was extracted using TRIzol solution (Invitrogen, Carlsbad, CA, USA). cDNA was synthesized with 0.5 µg RNA using PrimeScript™ 1st strand cDNA Synthesis kit (Takara, Tokyo, Japan) according to the manufacturer’s instructions. Quantitative real-time PCRs were conducted using StepOnePlus Real-time PCR System (Applied Biosystems, Carlsbad, CA, USA) and Thunderbird SYBR Qpcr mix (Toyobo, Osaka, Japan). The thermal cycling conditions consisted of 95 °C for 5 min, 40 cycles of 95 °C for 10 s, 60 °C for 20 s and 72 °C for 10 s. Primers used were as follows: fibronectin-sense 5′-ACAACA CCG AGG TGA CTG AGA C-3′, fibronectin-antisense 5′-GGA CAC AAC GAT GCT TCC TGA G-3′, COL1A1 (collagen type-1)-sense 5′-GAT TCC CTG GAC CTA AAG GTG C-3′, COL1A1-antisense 5′-AGC CTC TCC ATC TTT GCC AGC A-3′, MMP-1-sense 5′-ATG AAG CAG CCC AGA TGT GGA G-3′, MMP-1-antisense 5′-TGG TCC ACA TCT GCT CTT GGC A-3′, MMP-2-sense 5′-AGC GAG TGG ATG CCG CCT TTA A-3′, MMP-2-antisense 5′-CAT TCC AGG CAT CTG CGA TGA G-3′, MMP-3-sense 5′-CAC TCA CAG ACC TGA CTC GGT T-3′, MMP-3-antisense 5′-AAG CAG GAT CAC AGT TGG CTG G-3′, MMP-10-sense 5′-TCC AGG CTG TAT GAA GGA GAG G-3′, MMP-10-antisense 5′-GGT AGG CAT GAG CCA AAC TGT G-3′. All mRNA levels were normalized to β-actin.

### 4.8. Animals

ICR male mice (7 weeks old, 30–32 g) were purchased from ORIENT BIO Inc. (Gyeonggi-do, Republic of Korea). The mice were randomly separated in the individual cages to minimize wound disruption at 20 ± 5 °C, humidity 55 ± 10%, light and dark cycles of 12 h, and were allowed standard laboratory chow and water ad libitum. All procedures of animal experiments were approved by the Institutional Animal Care and Use Committee of Yonsei University in Korea (permit number: IACUC-A-20407-1876-02). At the end of the experiments, the mice were sacrificed by carbon dioxide inhalation.

### 4.9. In Vivo Wound Healing Assay

ICR male mice were anesthetized with isoflurane and maintained in the prone position on a temperature-controlled heating pad. The dorsal hair was removed using an electric clipper followed by depilatory cream application. The shaved area was disinfected with povidone-iodine solution and subsequently cleansed with 70% isopropyl alcohol to minimize potential cutaneous irritation. For wound creation, the dorsal skin between the scapulae was pinched along the midline and laterally positioned to ensure uniform distribution of the skin fold. Full-thickness excisional wounds were created using a sterile 6 mm disposable biopsy punch (Kai Industries Co., Ltd., Gifu, Japan). The mice were randomly divided into four groups (*n* = 6 per group): (1) vehicle control (vaseline containing 1% DMSO), (2) gestodene (2 mg/g vaseline), (3) vorapaxar (0.6 mg/g vaseline), and (4) combination treatment (gestodene 2 mg + vorapaxar 0.6 mg/g vaseline).

The designated treatments were topically applied to the wound sites once daily for 8 consecutive days. At each application, a standardized amount of each formulation was applied to ensure complete coverage of the wound surface. Wound closure was monitored by digital photography at a standardized height with a ruler for scale calibration. Images were captured on the day of injury and every subsequent 48 h. Wound areas were measured and analyzed using ImageJ software (version 1.53e, National Institutes of Health, Bethesda, MD, USA). The percentage of the wound area was calculated in relation to the initial area of the wound.

### 4.10. Masson’s Trichrome Staining

The dorsal wound tissues from ICR mice were fixed in 4% paraformaldehyde. The formalin-fixed tissue specimens were embedded in paraffin, cut into 4-μm-thick sections and stained with Masson’s trichrome (Sigma-Aldrich, St. Louis, MO, USA) according to the manufacturer’s instructions. Images were acquired using Zeiss Axio Scan.Z1 (Carl Zeiss Microscopy, Marly-le-Roi, France). For quantitative analysis of dermal collagen deposition, images were captured from the central portion of the wound site, excluding the wound margins, and a consistent region of interest (ROI) selection criterion was applied across all samples. The relative collagen-positive area (blue-stained area) was measured using the color deconvolution plugin function of ImageJ software (version 1.53e, National Institutes of Health, Bethesda, MD, USA) as described by Leite et al. [41]. The results were expressed as the percentage of collagen-positive area relative to the total tissue area.

### 4.11. Data and Statistical Analysis

All experiments were independently repeated a minimum of three times in a randomized manner. Data are expressed as mean ± SEM from ‘n’ independent experiments conducted in triplicate. Statistical comparisons were performed using unpaired or paired Student’s *t*-test, or one-way analysis of variance (ANOVA) with Tukey’s post hoc test for multiple comparisons, as appropriate. A *p*-value of less than 0.05 was considered statistically significant. Error bars represent the standard error of the mean. Concentration-response curves were generated and analyzed using GraphPad Prism 5.0 (GraphPad Software, San Diego, CA, USA).

## Data Availability

The data presented in this study are available on request from the corresponding author.

## Supplementary Materials

The following supporting information can be downloaded at: https://www.mdpi.com/article/10.3390/ijms27125502/s1.

## Abbreviations

PAR1	Protease-activated receptor 1
PAM	Positive allosteric modulator
GPCR	G protein-coupled receptor
APC	Activated Protein C
MAPK	Mitogen-activated protein kinase
PAR1-AP	PAR1-activating peptide
ECM	Extracellular matrix
HaCaT	Human keratinocyte
MMP	Matrix metalloproteinase
TRB	Thrombin
COL1A1	Collagen type I alpha 1
PAR1-KO	PAR1 knockout
HDF	Human dermal fibroblast

## References

[1] George Broughton I., Janis J.E., Attinger C.E. (2006). Wound healing: An overview. Plast. Reconstr. Surg..

[2] Guo S.A., DiPietro L.A. (2010). Factors affecting wound healing. J. Dent. Res..

[3] Black J., Baharestani M.M., Cuddigan J., Dorner B., Edsberg L., Langemo D., Posthauer M.E., Ratliff C., Taler G. (2007). National Pressure Ulcer Advisory Panel’s updated pressure ulcer staging system. Adv. Ski. Wound Care.

[4] Lavery L.A., Armstrong D.G., Harkless L.B. (1996). Classification of diabetic foot wounds. J. Foot Ankle Surg..

[5] Menke N.B., Ward K.R., Witten T.M., Bonchev D.G., Diegelmann R.F. (2007). Impaired wound healing. Clin. Dermatol..

[6] Lin A., Hokugo A., Nishimura I. (2010). Wound closure and wound management: A new therapeutic molecular target. Cell Adhes. Migr..

[7] Velnar T., Bailey T., Smrkolj V. (2009). The wound healing process: An overview of the cellular and molecular mechanisms. J. Int. Med. Res..

[8] Schultz G.S., Chin G.A., Moldawer L., Diegelmann R.F. (2011). 23 principles of wound healing. Mechanisms of Vascular Disease: A Reference Book for Vascular Specialists.

[9] Strukova S.M., Dugina T.N., Chistov I.V., Lange M., Markvicheva E.A., Kuptsova S., Zubov V.P., Glusa E. (2001). Immobilized thrombin receptor agonist peptide accelerates wound healing in mice. Clin. Appl. Thromb./Hemost..

[10] Mosnier L.O., Sinha R.K., Burnier L., Bouwens E.A., Griffin J.H. (2012). Biased agonism of protease-activated receptor 1 by activated protein C caused by noncanonical cleavage at Arg46. Blood J. Am. Soc. Hematol..

[11] Austin K.M., Covic L., Kuliopulos A. (2013). Matrix metalloproteases and PAR1 activation. Blood.

[12] Gieseler F., Ungefroren H., Settmacher U., Hollenberg M.D., Kaufmann R. (2013). Proteinase-activated receptors (PARs)–focus on receptor-receptor-interactions and their physiological and pathophysiological impact. Cell Commun. Signal..

[13] Vu T.-K.H., Hung D.T., Wheaton V.I., Coughlin S.R. (1991). Molecular cloning of a functional thrombin receptor reveals a novel proteolytic mechanism of receptor activation. Cell.

[14] Rohani M.G., DiJulio D.H., An J.Y., Hacker B.M., Dale B.A., Chung W.O. (2010). PAR1-and PAR2-induced innate immune markers are negatively regulated by PI3K/Akt signaling pathway in oral keratinocytes. BMC Immunol..

[15] Riewald M., Petrovan R.J., Donner A., Mueller B.M., Ruf W. (2002). Activation of endothelial cell protease activated receptor 1 by the protein C pathway. Science.

[16] Seo Y., Heo Y., Jo S., Park S.H., Lee C., Chang J., Jeon D.K., Kim T.G., Han G., Namkung W. (2021). Novel positive allosteric modulator of protease-activated receptor 1 promotes skin wound healing in hairless mice. Br. J. Pharmacol..

[17] Wang L., Luo J., He S. (2007). Induction of MMP-9 release from human dermal fibroblasts by thrombin: Involvement of JAK/STAT3 signaling pathway in MMP-9 release. BMC Cell Biol..

[18] Coughlin S.R. (2005). Protease-activated receptors in hemostasis, thrombosis and vascular biology. J. Thromb. Haemost..

[19] Trejo J. (2003). Protease-activated receptors: New concepts in regulation of G protein-coupled receptor signaling and trafficking. J. Pharmacol. Exp. Ther..

[20] Kuliopulos A., Covic L., Seeley S.K., Sheridan P.J., Helin J., Costello C.E. (1999). Plasmin desensitization of the PAR1 thrombin receptor: Kinetics, sites of truncation, and implications for thrombolytic therapy. Biochemistry.

[21] Foster D.J., Conn P.J. (2017). Allosteric Modulation of GPCRs: New Insights and Potential Utility for Treatment of Schizophrenia and Other CNS Disorders. Neuron.

[22] Wold E.A., Zhou J. (2018). GPCR Allosteric Modulators: Mechanistic Advantages and Therapeutic Applications. Curr. Top. Med. Chem..

[23] Heo Y., Yang E., Lee Y., Seo Y., Ryu K., Jeon H., Namkung W. (2022). GB83, an Agonist of PAR2 with a Unique Mechanism of Action Distinct from Trypsin and PAR2-AP. Int. J. Mol. Sci..

[24] Barry G.D., Suen J.Y., Le G.T., Cotterell A., Reid R.C., Fairlie D.P. (2010). Novel agonists and antagonists for human protease activated receptor 2. J. Med. Chem..

[25] Park S.-H., Heo Y., Kwon I., Jo S., Jeon H., Lee Y., Kim J., Heo J.H., Namkung W. (2024). Gestodene, a novel positive allosteric modulator of PAR1, enhances PAR1-mediated human platelet aggregation. Front. Pharmacol..

[26] Heo Y., Jeon H., Namkung W. (2022). PAR4-Mediated PI3K/Akt and RhoA/ROCK Signaling Pathways Are Essential for Thrombin-Induced Morphological Changes in MEG-01 Cells. Int. J. Mol. Sci..

[27] Salo T., Mäkelä M., Kylmäniemi M., Autio-Harmainen H., Larjava H. (1994). Expression of matrix metalloproteinase-2 and-9 during early human wound healing. Lab. Investig. A J. Tech. Methods Pathol..

[28] Kandhwal M., Behl T., Singh S., Sharma N., Arora S., Bhatia S., Al-Harrasi A., Sachdeva M., Bungau S. (2022). Role of matrix metalloproteinase in wound healing. Am. J. Transl. Res..

[29] Worthen C.A., Cui Y., Orringer J.S., Johnson T.M., Voorhees J.J., Fisher G.J. (2020). CD26 Identifies a Subpopulation of Fibroblasts that Produce the Majority of Collagen during Wound Healing in Human Skin. J. Investig. Dermatol..

[30] Lenselink E.A. (2015). Role of fibronectin in normal wound healing. Int. Wound J..

[31] Kaplan B. (1995). Desogestrel, norgestimate, and gestodene: The newer progestins. Ann. Pharmacother..

[32] Ashburn T.T., Thor K.B. (2004). Drug repositioning: Identifying and developing new uses for existing drugs. Nat. Rev. Drug Discov..

[33] Jourdan J.-P., Bureau R., Rochais C., Dallemagne P. (2020). Drug repositioning: A brief overview. J. Pharm. Pharmacol..

[34] Ubl J.J., Sergeeva M., Reiser G. (2000). Desensitisation of protease-activated receptor-1 (PAR-1) in rat astrocytes: Evidence for a novel mechanism for terminating Ca^2+^ signalling evoked by the tethered ligand. J. Physiol..

[35] Burford N.T., Clark M.J., Wehrman T.S., Gerritz S.W., Banks M., O’Connell J., Traynor J.R., Alt A. (2013). Discovery of positive allosteric modulators and silent allosteric modulators of the μ-opioid receptor. Proc. Natl. Acad. Sci. USA.

[36] Moran S.P., Dickerson J.W., Cho H.P., Xiang Z., Maksymetz J., Remke D.H., Lv X., Doyle C.A., Rajan D.H., Niswender C.M. (2018). M(1)-positive allosteric modulators lacking agonist activity provide the optimal profile for enhancing cognition. Neuropsychopharmacology.

[37] Leger A.J., Covic L., Kuliopulos A. (2006). Protease-Activated Receptors in Cardiovascular Diseases. Circulation.

[38] Fan M., Fan X., Lai Y., Chen J., Peng Y., Peng Y., Xiang L., Ma Y. (2024). Protease-Activated Receptor 2 in inflammatory skin disease: Current evidence and future perspectives. Front. Immunol..

[39] Lee S.E., Jeong S.K., Lee S.H. (2010). Protease and protease-activated receptor-2 signaling in the pathogenesis of atopic dermatitis. Yonsei Med. J..

[40] Park M.K., Cho M.K., Kang S.A., Park H.-K., Kim Y.S., Kim K.U., Ahn S.C., Kim D.-H., Yu H.S. (2011). Protease-activated receptor 2 is involved in Th2 responses against Trichinella spiralis infection. Korean J. Parasitol..

[41] Leite M.N., Leite S.N., Caetano G.F., Andrade T.A.M.d., Fronza M., Frade M.A.C. (2020). Healing effects of natural latex serum 1% from Hevea brasiliensis in an experimental skin abrasion wound model. An. Bras. Dermatol..

