# Supplementary material for: Gestodene Accelerates Cutaneous Wound Healing via PAR1-Selective Positive Allosteric Modulation
Source: Int J Mol Sci. 2026 Jun 18;27(12):5502. doi: 10.3390/ijms27125502 (PMC13300339; doi:10.3390/ijms27125502)
Supplement: Supplementary file 1 [file ijms-27-05502-s001.zip › ijms-4327158-supplementary.pdf]

Supplementary Materials Data

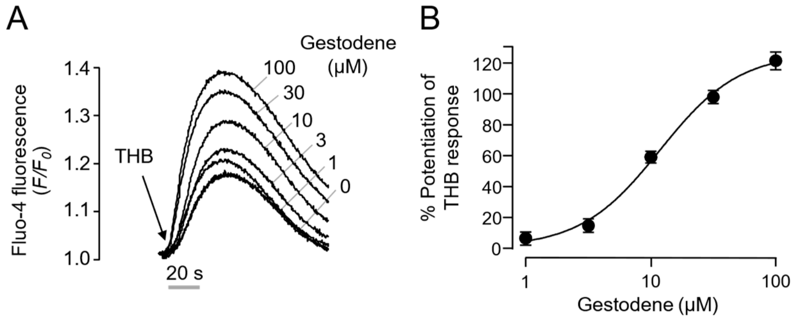

Figure S1. Enhancement of thrombin-induced intracellular calcium signaling by gestodene in HaCaT cells. (A) The indicated concentrations of gestodene were applied 10 min prior to thrombin (THB) (0.4 Unit/mL) treatment. Intracellular calcium responses are expressed as Fluo-4 fluorescence intensity (F/F₀). (B) Dose–response curve of potentiation of thrombin-induced calcium responses by gestodene in HaCaT cells. Data are expressed as the percentage potentiation above thrombin (0.4 Unit/mL) alone (mean ± S.D., n = 4).

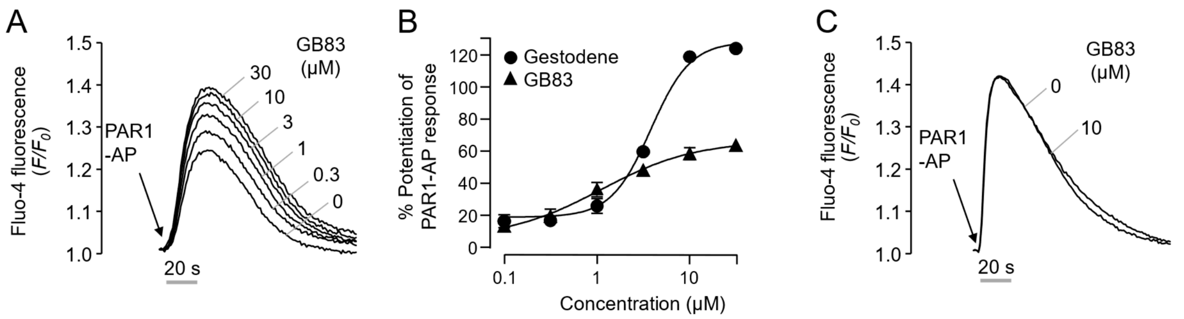

Figure S2. Comparison of PAR1-AP-induced intracellular calcium potentiation by GB83 and gestodene in HaCaT cells. (A) The indicated concentrations of GB83 were applied 10 min prior to PAR1-AP (20 μM) treatment. (B) Dose–response curves of potentiation of PAR1-AP (20 μM)-induced calcium responses by gestodene and GB83 in HaCaT cells. Gestodene data were obtained from Figure 3A. Data are expressed as the percentage potentiation above PAR1-AP (20 μM) alone. (C) Effect of GB83 (10 μM) on PAR1-AP (100 μM)-induced maximal calcium response. (mean ± S.D., n = 4).

Table S1. Statistical comparison of wound closure at each time point

Day	Vorapaxer vs. Vehicle	Getodene vs. Vehicle	Getodene +Vorapaxer vs. Vehicle	Getodene +Vorapaxer vs. Gestodene
2	0.804	0.063	0.907	0.139
4	0.948	0.064	0.758	0.105
6	0.214	0.011	0.700	0.007
8	0.634	<0.001	0.373	<0.001

Table S1. Exact p values for pairwise comparisons of relative wound area at each time point. Statistical analysis was performed using one-way ANOVA followed by Tukey's post hoc test. Values less than 0.001 are expressed as <0.001.
